# Assessment of Respiratory Rate and Simulated Apnea Utilizing the PneumoWave Biosensor: In Vitro and In Vivo Validation

**DOI:** 10.3390/bios16050256

**Published:** 2026-05-01

**Authors:** Burcu Kolukisa Birgec, Beyza Toprak, Alexander Balfour Mullen

**Affiliations:** Strathclyde Institute of Pharmacy and Biomedical Sciences, University of Strathclyde, Glasgow G4 0RE, UK; burcu.kolukisa@strath.ac.uk (B.K.B.); beyza.toprak.2022@uni.strath.ac.uk (B.T.)

**Keywords:** biosensor, respiratory rate monitoring, apnea, validation

## Abstract

Accurate monitoring of respiratory rates is critical for early detection of a range of clinical conditions. However, standard manual counting or inadequate clinical monitoring often fails to provide reliable measurements. This study evaluated and validated the PneumoWave biosensor for respiratory rate measurement across a broad physiological range and different body postures (45°, 90°, and 180°) in both in vitro and in vivo settings. In vitro validation was performed using a SimMan ALS manikin operated at respiratory settings of 6–30 breaths per minute, with 10 s periods of simulated apnea. In vivo validation involved 20 healthy volunteers performing metronome-guided breathing while wearing bilateral PneumoWave biosensors. In vitro results demonstrated an excellent correlation between biosensors and manikin respiratory settings and captured all apnea events (r = 0.99, ICC = 0.99). In vivo findings showed good agreement with direct observational count (r = 0.99, R^2^ = 0.99, ICC = 0.99), with 97% of apnea events captured by both devices in all positions. Body postures had no significant impact on biosensor accuracy. These findings demonstrate that the PneumoWave biosensor provides accurate and reliable respiratory monitoring and supports its potential as a robust, non-invasive tool for continuous clinical and remote patient monitoring.

## 1. Introduction

Respiratory rate (RR) is an important vital sign that responds rapidly to physiological and environmental stressors, including exercise, emotion, and fatigue. Respiratory rate is often the first parameter to deviate during clinical deterioration [1,2,3,4]. The ability to accurately monitor respiratory rate is therefore essential to support diagnosis in a range of clinical conditions, e.g., congestive heart failure, pulmonary embolism, pneumonia, Chronic Obstructive Pulmonary Disease (COPD), asthma and sepsis [1,5,6,7]. Previous studies have established that respiratory rate is a superior discriminator of clinical instability compared to heart rate or blood pressure, with rates exceeding 24–27 breaths per minute (BPM) serving as a critical predictor of adverse events such cardiac arrest and even mortality up to 24 h in advance [6,8,9,10]. The predictive value of respiratory rate as a sensitive marker of clinical decline was stark during the COVID-19 pandemic and highlighted the importance of reliable RR monitoring in both clinical and home-care settings [11,12,13,14]. 

In routine clinical practice, respiratory rate is assessed by counting visible chest excursion over one minute while the patient is at rest, alongside qualitative observations of consciousness level, breathing pattern and effort, skin colour, and symmetry of chest expansion. However, manual respiratory rate assessment, despite being widely used as a clinical standard, has been shown to be inconsistently applied and susceptible to measurement error in routine practice [15,16,17,18]. Although capnography is commonly used in intensive care settings, it is invasive, resource-intensive, and not readily scalable to general ward or long-term monitoring contexts [5,6,11,15,19,20,21].

To address the limitations associated with manual counting, there is growing demand for wearable devices capable of continuously monitoring respiratory rate [1,22,23]. Although several systems are now commercially available, their performance remains variable, with reported limitations including reduced accuracy, restricted operational respiratory ranges, and sensitivity to body position [24,25,26,27,28]. 

Among these, accelerometer-based systems have been widely investigated as a means of capturing thoracic motion associated with breathing. A range of signal processing strategies have been proposed for accelerometer-based respiratory monitoring, including time–domain, frequency–domain, and time–frequency approaches [29,30]. In practice, these methods are often combined with preprocessing steps such as filtering and signal smoothing to reduce noise and improve respiratory signal quality [30,31,32]. However, performance remains dependent on signal quality, processing choices, and susceptibility to motion artefacts.

Despite these advances, many studies have focused on algorithm development using raw sensor data under controlled conditions. However, algorithm-level performance does not necessarily translate to real-world device performance, where respiratory rate is derived from device-generated signals processed within proprietary frameworks. This distinction is particularly relevant for wearable systems intended for clinical and remote monitoring, where end users rely on device-derived outputs rather than custom signal processing pipelines.

In this context, the present study adopts a validation-focused approach by evaluating respiratory rate estimation based on device-generated signals, rather than introducing or optimizing a new signal processing algorithm. By preserving the manufacturer’s processing framework (manual counting) this study aims to provide a more representative assessment of biosensor performance under controlled but physiologically relevant conditions.

The PneumoWave biosensor is a UKCA Class I wearable device that enables continuous, non-invasive respiratory monitoring and provides researcher-level access to combined accelerometer-derived waveform data via a dashboard system [33,34]. The analyzed combined data are derived from raw accelerometer signals via a proprietary and undisclosed processing framework designed by the manufacture [35]. This present study established in vitro and in vivo validation of the PneumoWave biosensor using adult manikin and healthy volunteers, respectively. Device performance was evaluated across a broad range of target respiratory rates (6–30 breaths per minute) and body postures, including periods of simulated apnea.

## 2. Materials and Methods

### 2.1. In Vitro Validation

The PneumoWave biosensor hardware and software were used as previously described [33,34]. Two identical PneumoWave biosensors (PW010, PneumoWave, Maxim Park, UK) were positioned simultaneously on the upper left and right chest of an adult manikin (SimMan ALS, Laerdal Medical, London, UK), approximately 6 cm inferior to the midpoint of each clavicle, to enable bilateral signal acquisition. The manikin was operated using Laerdal ALS Software for SimPad (v2.0.0.122, Laerdal Medical, London, UK).

Predefined simulated respiratory rates of 6, 10, 15, 20, 25, and 30 breaths per minute (BPM) were generated and recorded by both biosensors. These values were selected to represent clinically relevant low, normal, and elevated respiratory rate ranges. For each respiratory rate, recordings were obtained for three minutes and repeated five times (*n* = 5). Experiments were conducted across three body postures: semi-recumbent (45°), upright (90°), and supine (180°). Simulated apnea episodes were introduced by setting the manikin respiratory rate to 0 BPM for 10 s.

### 2.2. In Vivo Validation

#### 2.2.1. Participants and Ethical Considerations

All relevant study material and ethical approval were obtained from the University Ethics Committee (Reference UEC24/91, University of Strathclyde, February 2025). All participants provided written informed consent prior to participation. Volunteers were informed of the study objectives, procedures, and potential risks, and were advised that participation was voluntary and that they could withdraw at any time without consequence. Prior to recruitment, all participants completed a pre-screening questionnaire and a truncated paced breathing assessment (3 min at both 6 and 30 BPM) to determine eligibility (see Supplementary Material-Recruitment Information). Following screening, 20 eligible volunteers were enrolled. PRISMA flow (Figure S1) is represented in Supplementary Materials. All data were collected, stored, and processed in full accordance with the General Data Protection Regulation (GDPR; Regulation (EU) 2016/679 of the European Parliament and of the Council of 27 April 2016). Volunteer identities were fully anonymized prior to analysis, and all research data were stored on a secured university cloud.

#### 2.2.2. Experimental Protocol

Two PneumoWave biosensors were positioned on the upper left and right chest walls of each participant, approximately 6 cm inferior to the midpoint of the clavicle. Participants performed paced breathing guided by a digital metronome application (Metronome Beats, Stonekick Ltd., London, UK, v6.10.1) delivered through headphones.

To ensure balanced exposure across the full respiratory spectrum and to prevent systematic allocation of only low or high breathing rates to specific individuals, respiratory rates were grouped into five predefined categories: bradypnea (6–10 breaths per minute), normal (11–16 breaths per minute), normal/mild tachypnea (17–22 breaths per minute), moderate tachypnea (23–28 breaths per minute), and severe tachypnea (29–30 breaths per minute). Each participant was randomly assigned, using a random number generator (www.random.org, accessed 1 August 2025), to one specific respiratory rate within each pre-defined category, resulting in five recordings per participant covering the full respiratory range.

Each recording lasted 4 min and 20 s and consisted of a 20 s stabilization period, 3 min of metronome-guided breathing at the assigned rate, 10 s of voluntary breath-hold to simulate apnea, and 50 s of spontaneous breathing. Recordings were repeated adopting three body postures: semi-recumbent (45°), upright (90°), and supine (180°). Participants rested for 2–5 min between measurements to minimize fatigue and ensure respiratory stabilization.

Experiments were conducted in a single-blind manner, with the researcher performing manual respiratory counting unaware of the assigned paced-breathing rate. Apnea duration was adjudicated by two researchers, with one identifying thoracic motion cessation through direct observation and the second quantifying event duration using synchronized digital timing.

### 2.3. Data Processing and Statistical Analysis 

**Figure 1:**
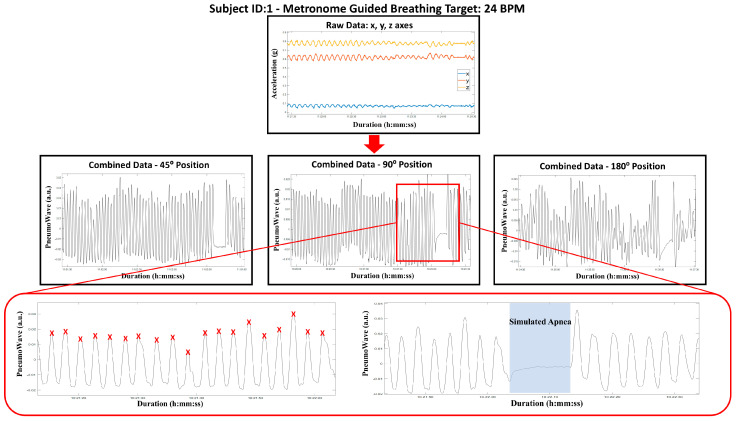
Representative workflow of respiratory signal processing and event identification. Raw triaxial (x, y, and z) accelerometer data captured by the PneumoWave biosensor. The combined accelerometer waveform, generated by the manufacturer and derived from the raw axes, is visualized, and manual waveform peak-to-peak counting (red cross) and a 10 s simulated apnea event (area highlighted in blue) are evaluated for each posture. a.u.: arbitrary unit, g: acceleration.

Data were transmitted via Bluetooth to a linked tablet device (LTE model, Android 11, Samsung, Suwon, Republic of Korea) running the PneumoWave DC application (v1.0.8, PneumoWave, Maxim Park, UK) and subsequently exported in CSV format from the PneumoWave Cloud Investigator Dashboard (PneumoWave, Maxim Park, UK) for analysis. Accelerometer data from both in vitro and in vivo experiments were exported in CSV format from the PneumoWave Cloud Investigator Dashboard. Analysis was performed on the manufacturer-generated combined accelerometer signal, derived from the triaxial (x, y, z) sensor data. The integrated waveform was visualized and analyzed using MATLAB (Version R2023a, MathWorks, Natick, MA, USA) for the manual respiratory cycle identification (Figure 1). No additional filtering or signal transformation was applied to the combined accelerometer data prior to analysis.

The data analysis was categorized into two primary sections: respiratory rate (RR) accuracy and simulated apnea detection. The performance of each biosensor was evaluated against the established reference standards to determine clinical accuracy. Furthermore, inter-device reliability was assessed to ensure consistency between the Left- (L) and Right-sided (R) sensors across all body positions.

#### Respiratory Rate and Simulated Apnea Analysis

Biosensor-derived respiratory rate was quantified using the Manual Average Visual Count (MAVC) method, as previously defined [33]. Following waveform visualization, respiratory cycles were manually identified through direct inspection of the combined signal with continuous windowing equal to the entire trial duration. A respiratory cycle was defined based on consistent waveform periodicity, typically corresponding to identifiable peak-to-peak intervals associated with thoracic expansion and contraction by two trained researchers. Recordings were divided between researchers, and ambiguous segments cross-checked to ensure consistency in cycle identification. For in vitro validation, the Laerdal ALS system was accepted as the reference standard. For in vivo validation, Direct Observation Count (DOC) served as the reference standard. A trained researcher directly observed and counted visible chest excursions over a three-minute period, with the total divided by three to obtain an average breaths-per-minute value. To verify participant adherence to the paced-breathing protocol, the correlation between the target metronome rates and the researcher’s Direct Observation Count (DOC) was evaluated to ensure the fidelity of the reference standard.

Agreement of respiratory rate between biosensor-derived data and reference standard was evaluated using Pearson’s correlation analysis (r) and the coefficient of determination (R^2^) to quantify the proportion of variance between the biosensor measurements and the reference standards. Bland–Altman plots with 95% confidence intervals were also utilized to assess bias and limits of agreement (LoA). The Intraclass Correlation Coefficient (ICC) was calculated to assess consistency and agreement between methods, utilizing a two-way mixed-effects model with a single-measure absolute agreement definition. Biosensor accuracy was further quantified using mean absolute error (MAE). A predefined ±2 BPM threshold was applied to assess clinical acceptability. This threshold was selected to align with commonly applied evaluation criteria in respiratory monitoring research and with tolerances reported in prior wearable device studies [5,36]. This threshold is also consistent with previously published evaluations of the PneumoWave system, supporting methodological continuity [37]. Inter-device reliability between the Left (L) and Right (R) biosensor placements was evaluated using the same statistical framework as the accuracy analysis. 

Simulated apnea events were defined in accordance with the American Academy of Sleep Medicine (AASM) criteria, which is breath cessation a minimum duration of 10 s [38]. In this study, apnea was characterized by a ≥90% reduction in sensor-derived thoracic motion signal according to AASM criteria. For both in vitro and in vivo validation, this 10 s breath cessation was established as the reference standard, and all subsequent manual analyses were conducted based on this predefined 90% signal reduction threshold. In vitro and in vivo settings, accuracy (relative to the 10 s reference) were quantified through mean difference, MAE, and Bland–Altman plots to ensure consistency in event detection while in vivo experiment accuracy and inter-device reliability evaluated across both sensors. All statistical analyses were performed using SPSS (Version 29.0.1.0, IBM Corp., Armonk, NY, USA). Continuous data are presented as mean ± standard deviation (SD) with 95% confidence intervals (CI). Statistical significance was defined as *p* < 0.05.

## 3. Results

### 3.1. In Vitro Experiment 

#### 3.1.1. Respiratory Rate 

**Figure 2:**
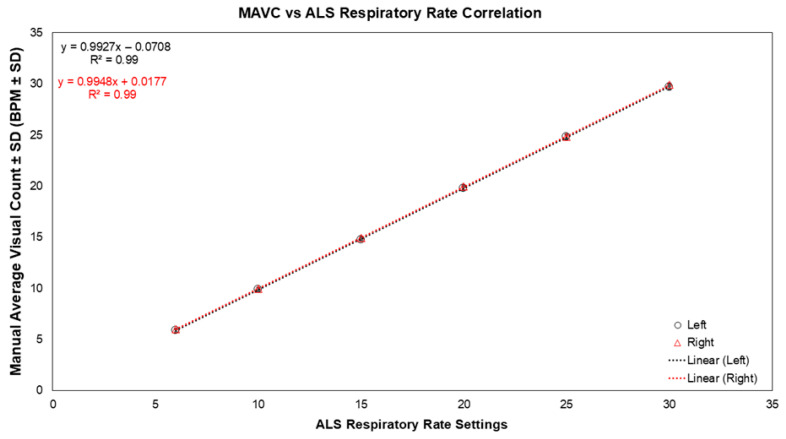
Scatterplot of mean Manual Average Visual Count (MAVC) from the Left and Right PneumoWave biosensors across all body positions plotted against Laerdal ALS manikin respiratory rate settings (*n* = 15). Circles represent the Left side PneumoWave biosensor and triangles represent the Right side PneumoWave biosensor.

Left and right PneumoWave biosensors demonstrated near-perfect correlation and agreement with the Laerdal ALS respiratory settings across all body postures (r = 0.99, R^2^ = 0.99, 95% CI 0.999–1.000, *p* < 0.001; Figure 2). ICC values were 0.99 (95% CI 0.996–1.000) across postures. Standard deviations across respiratory rates ranged from 0.15 to 0.23 BPM for the left sensor and 0 to 0.21 BPM for the right sensor, reflecting high measurement reproducibility within the mechanical simulation. Bland–Altman analysis demonstrated low percentage bias across postures. For the left biosensor, mean bias ranged from 1.06% to 1.59%, with 95% limits of agreement (LoA) between −2.11% and 5.30%. For the right biosensor, mean bias ranged from 0.17% to 0.55%, with LoA between −1.59% and 2.68%. Across all postures, one data point at 90° and four at 180° (Left sensor) and six total observations (Right sensor) fell outside the 95% LoA.

No meaningful differences in respiratory rate agreement were observed across 45°, 90°, and 180° three body postures. Inter-device comparison demonstrated small mean differences between left and right sensors of −0.09 ± 0.17 BPM, −0.18 ± 0.19 BPM, and −0.11 ± 0.22 BPM at 45°, 90°, and 180°, respectively. Percentage bias between devices ranged from −0.52% to −1.18%, with the broadest 95% LoA remaining within −5.29% and 2.93%.

#### 3.1.2. Simulated Apnea

**Figure 3:**
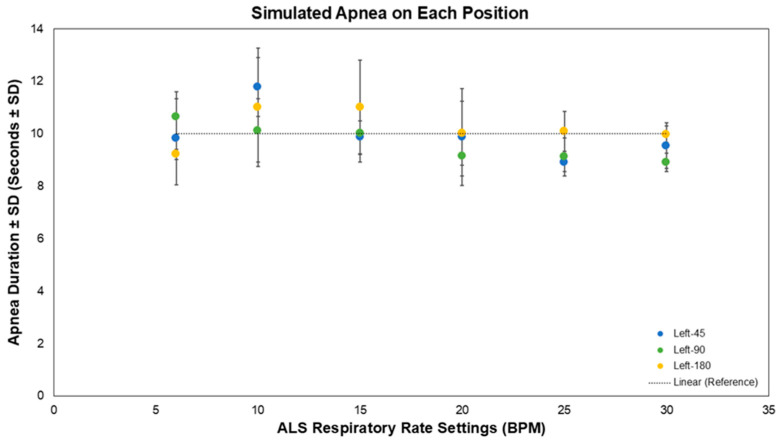
Simulated 10 s apnea on the manikin at 6–30 BPM on each posture (*n* = 5). Circles represent the Left PneumoWave device. Blue represents the for 45° posture; green represents 90° posture and yellow represents 180° posture.

All simulated 10 s apnea events were successfully detected across the respiratory range of 6–30 breaths per minute (Figure 3). The biosensor consistently identified cessation and resumption of respiratory motion. Mean detected apnea duration for the left sensor was 9.97 ± 1.43 s (45°), 9.66 ± 1.01 s (90°), and 10.22 ± 1.37 s (180°). Performance remained stable across body posture.

### 3.2. In Vivo Validation

#### 3.2.1. Patient Demographics

Demographic characteristics (age, weight, height, and body mass index (BMI)) of the 20 healthy volunteers are summarized in Table 1. Fifty percent of volunteers were female. The ethnicity of volunteers, reported as categories used in UK patient demographic data and the Office of National Statistics, were: White/Caucasian (40%), Asian/Asian British (10%) and Other (50%).

#### 3.2.2. Respiratory Rate Analysis

**Figure 4:**
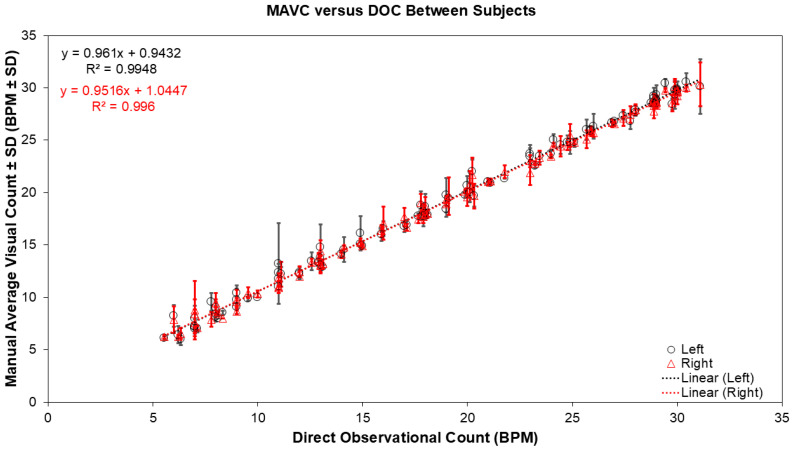
The correlation between the pooled mean Manual Average Visual Count (MAVC) (±SD), derived from both Left (circles) and Right (triangles) PneumoWave biosensors across all three body postures, and the Direct Observation Count (DOC). Data points represent the average respiratory rate for each subject across the 45°, 90°, and 180° postures.

Correlation and absolute agreement between metronome target rates and Direct Observation Count (DOC) was excellent in all postures (r = 0.99, ICC = 0.99, 95% CI 0.998–1.000, *p* < 0.001), confirming DOC as a reliable reference. Both Left and Right biosensors demonstrated strong correlation with DOC across all body postures (r ≥ 0.99, *p* < 0.001) (Figure 4). ICC values remained 0.99 across postures. 

**Figure 5:**
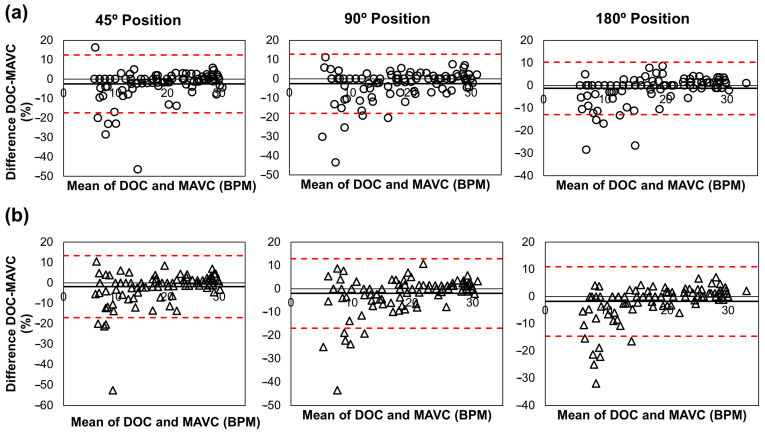
Bland–Altman plots showing the agreement between DOC and the (**a**) Left and (**b**) Right biosensors across the 6–30 BPM range. Circles (**top**) and triangles (**bottom**) represent individual data points for each sensor, respectively. Solid black lines indicate the mean bias, and dashed red lines represent the 95% limits of agreement (±1.96 SD). DOC: Direct Observation Count; MAVC: Manual Average Visual Count.

Mean differences between biosensor-derived respiratory rate and DOC ranged from −0.04 to −0.29 BPM, with mean absolute error (MAE) between 0.51 and 0.67 BPM. Bland–Altman analysis showed percentage bias ranging from −1.29% to −2.54% (Figure 5). The lower 95% LoA ranged from −17.32% to −12.91%, and the upper LoA ranged from 10.34% to 13.33% (Table 2, Section I).

#### 3.2.3. Influence of Body Posture and Sensor Placement

Body posture had no statistically significant effect on biosensor accuracy (*p* > 0.05). Inter-device reliability between left and right sensors was high across all positions (ICC ≥ 0.98) (Table 2). Mean inter-device differences were ≤0.14 BPM. Bland–Altman analysis of inter-device agreement showed percentage bias of 0.59%, 0.51%, and −0.53% at 45°, 90°, and 180°, respectively, with lower LoA between −19.01% and −14.24% and upper LoA between 13.18% and 20.19%. Data points were evenly distributed along the mean bias line. Bland–Altman figure (Figure S2) is reported in Supplementary Materials. 

#### 3.2.4. Simulated Periods of Apnea

**Figure 6:**
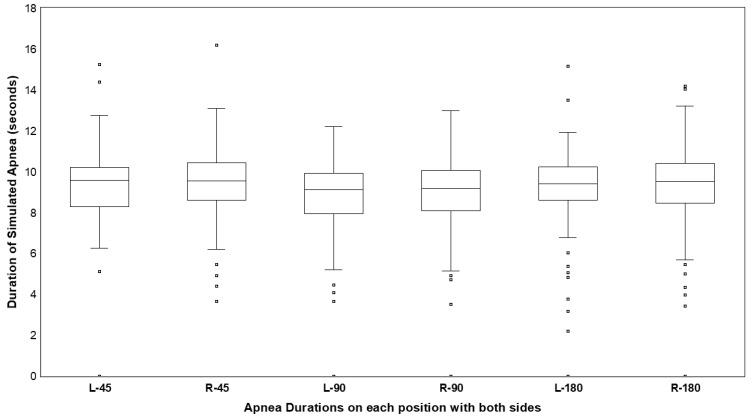
Box plots of the duration of simulated apnea on each position. Lower and upper fences are quartile 1 and 3 while the mean is between. Bars are showing maximum and minimum. Squares are outliers. L: PneumoWave biosensor on the left side, R: PneumoWave biosensor on the right side. 45: 45° sitting postures, 90: 90° sitting postures, 180: 180° postures.

Across 20 participants, five respiratory rates, and three body positions, 300 simulated apnea events were performed per device, resulting in 600 observations. Of these, 582 events (97.0%) were successfully detected, while 18 (3.0%) were not identified. Among detected events, 17 (2.83%) had recorded durations of less than 5 s, with the remainder meeting or exceeding the predefined 5 s threshold. Mean detected apnea duration ranged from 8.58 ± 2.35 s to 9.43 ± 1.76 s across postures (Figure 6; Table 3–Section I). Detection performance was not significantly influenced by body posture (*p* > 0.05). Inter-device differences in detected apnea duration ranged from −0.41 to −0.05 s across posture.

## 4. Discussion

### 4.1. Respiratory Rate Measurement

For in vitro manikin experiments, correlation and agreement between the biosensor-derived Manual Average Visual Count (MAVC) and predefined respiratory settings was near-perfect, confirming technical measurement accuracy. Importantly, this level of agreement was maintained in healthy volunteers, where natural breath-to-breath variability introduces greater physiological complexity.

Among the tested postures, the supine (180°) position demonstrated the lowest mean absolute error and minimal mean bias, with mean differences as low as −0.04 BPM in vivo. When evaluated using a ±2 BPM threshold, approximately 96–99% of measurements fell within acceptable agreement limits, with the highest proportion observed in the supine posture. From a signal-processing perspective, stable performance in the supine position indicates consistent thoracic motion capture despite altered gravitational vector alignment, supporting the suitability of accelerometer-based monitoring for sleep and bed-bound applications.

Overall, body posture did not significantly influence measurement accuracy (*p* > 0.05). Furthermore, no clinically meaningful differences were observed between left- and right-sided sensor placements. Inter-device agreement remained high (ICC ≥ 0.98), with minimal mean differences across postures. This symmetrical performance suggests flexibility in clinical placement without compromising measurement precision, which is advantageous for both ward-based and home monitoring contexts.

Notably, performance under the ±2 BPM threshold demonstrates precision beyond the broader ±3–5 BPM error margins commonly reported for non-invasive respiratory monitoring systems [39,40,41]. Several previously validated technologies have shown acceptable agreement but often within restricted respiratory ranges, shorter measurement duration or limited postural testing. For example, impedance-based respiratory belts have reported different limits of agreement depending on reference standard device [42], radar-based systems such as Somnofy have demonstrated moderate bias ranges [43], and consumer-grade wearable devices have shown moderate reliability coefficients (e.g., ICC ≈ 0.77 for Fitbit Charge 4) [44]. In contrast, the present study assessed randomized respiratory rates spanning bradypnea to tachypnea states and included evaluation across multiple body positions, including device reliability, providing a broader physiological validation framework. The sustained high agreement under these expanded conditions suggests stable accelerometer signal integration and reliable respiratory cycle extraction.

### 4.2. Apnea Detection

The biosensor demonstrated robust detection of simulated respiratory cessation. All apnea events were identified in vitro, and 97% were detected in vivo. The slightly shorter mean detected duration relative to the 10 s reference (approximately 8.6–9.4 s) observed in vivo, likely reflects physiological variability associated with voluntary breath-holding, including micro-movements at initiation and termination. Importantly, detection performance was not significantly influenced by body posture, and inter-device differences in measured duration were minimal.

Across the 600 in vivo apnea measurements, 94% of events lasting ≥ 5 s were successfully detected. This suggests reliable sensitivity to clinically relevant respiratory pauses and supports the technical feasibility of accelerometer-based apnea identification under controlled breath-hold conditions.

### 4.3. Clinical Context and Translational Relevance

Respiratory rate remains a sensitive physiological marker, yet continuous monitoring in routine practice is often limited by reliance on intermittent manual counting. Wearable accelerometer-based systems may address this gap by enabling continuous, non-invasive respiratory surveillance. The increased adoption of remote monitoring technologies during and after the COVID-19 pandemic has further emphasized the need for reliable wearable respiratory sensors [45].

The current findings are based upon prior technical development and proof-of-concept investigations involving opioid-related respiratory depression and pediatric sleep apnea [33,34,37,46]. Ongoing research initiatives, including investigations of COPD deterioration (Clinical Trial ID: NCT06419036), extend the potential application of the PneumoWave biosensor to chronic respiratory disease and high-risk populations.

In sleep medicine, night-to-night variability in obstructive sleep apnea (OSA) contributes to diagnostic misclassification rates of 20–50% following single-night recordings, with improved reliability observed after multi-night monitoring [47]. A wearable system capable of stable supine monitoring may therefore offer practical advantages for longitudinal respiratory profiling. Although home sleep apnea testing is not currently recommended for pediatric populations by the American Academy of Sleep Medicine (AASM) [48], multi-night physiological monitoring may support longitudinal assessment in selected clinical contexts. This capability is particularly significant for complex cases where laboratory-based polysomnography is not feasible or representative, such as in low-weight newborns or children with behavioural problems who may struggle with the invasive and tethered nature of traditional monitoring equipment.

### 4.4. Strengths

A key strength of this study is the structured validation design integrating controlled in vitro verification with physiological in vivo testing. Bilateral sensor placement enabled assessment of inter-device reproducibility. Evaluation across a wide respiratory range (6–30 BPM), including bradypnea and tachypnea states, strengthens the physiological relevance of the validation framework. The inclusion of simulated apnea events further supports evaluation of the device’s ability to detect respiratory cessation.

### 4.5. Limitations

Several limitations should be considered. First, this study was conducted under controlled, static conditions and therefore does not account for dynamic motion artefacts, which remain a primary challenge in accelerometer-based wearable respiratory monitoring. In real-world settings, body movement, posture transitions, and external disturbances can significantly affect signal quality and respiratory cycle detection, and these factors were not assessed in the present validation framework. 

Second, the in vivo cohort comprised a relatively small sample of healthy volunteers, limiting extrapolation to patients with altered respiratory mechanics, irregular breathing patterns, or increased susceptibility to motion artefacts. Furthermore, the absence of stratification across demographic and physiological variables, such as age and body mass index (BMI), restricts assessment of how individual characteristics may influence respiratory signal morphology and device performance.

Third, apnea events were simulated rather than spontaneous, and voluntary breath-holding introduces inherent physiological variability. Voluntary breath-holding involves conscious muscle control and micro-movements that do not fully replicate the involuntary neural dynamics of clinical apnea. These physiological differences, such as subtle tremors at the initiation or termination of the breath-hold, can interfere with precise threshold apnea detection compared to pathological respiratory cessation. 

Finally, respiratory rate extraction relied on manual waveform inspection, which may introduce observer-dependent variability despite structured cross-check procedures. However, this approach was intentionally adopted to establish a transparent and interpretable reference for validation based on device-generated signals, without introducing additional algorithmic bias. 

### 4.6. Future Directions

To extend these controlled validation findings, longer-term monitoring in real-world environments, including hospital wards and home settings, will be necessary to evaluate usability, motion robustness, and longitudinal signal stability. Further studies should assess performance in clinical populations such as individuals with COPD, sleep-disordered breathing, and acute respiratory compromise. Development and validation of automated respiratory rate and apnea detection algorithms will be important to enhance analytical efficiency and facilitate large-scale deployment. Future integration of automated cycle and apnea detection algorithms is warranted to enhance scalability and reduce observer dependency. Given the data transparency from the PneumoWave system, such advancements could support a semi-automated, clinician-supervised framework. This would significantly streamline respiratory rate and apnea estimation while ensuring that final clinical oversight still remains with the healthcare professional, thereby maintaining diagnostic reliability and transparency during real-world deployment.

## 5. Conclusions

The PneumoWave biosensor demonstrated high agreement with reference standards for respiratory rate measurement and reliable performance in simulated apnea detection across multiple body positions. Superior accuracy in the supine posture and absence of clinically meaningful left–right placement differences support its suitability for sleep-related and continuous respiratory monitoring applications. These findings provide a technically robust foundation for further clinical and real-world validation studies.

## Figures and Tables

**Table 1:** Demographic characteristics of healthy volunteer participants.

Variables	Mean ± SD (Range)
Age (years)	29.8 ± 4.2 (24–39)
Weight (kg)	75.2 ± 15.5 (51–102)
Height (cm)	170.7 ± 8.1 (152–183)
BMI (kg/m^2^)	25.6 ± 3.77 (17.2–32.9)

**Table 2:** Summary of the results of accuracy and inter-device reliability on each position. MAE: Mean Absolute Error, SD: Standard Deviation, CI: Confidence Interval, DOC: Direct Observational Count, MAVC-L: Manual Average Visual Count from PneumoWave biosensor on the Left side; MAVC-R: Manual Average Visual Count from PneumoWave biosensor on the Right side.

	45°		90°		180°	
	MAVC-L	MAVC-R	MAVC-L	MAVC-R	MAVC-L	MAVC-R
**I. Accuracy (DOC vs. MAVC)**						
Correlation (95% CI, *p* value)	0.99 (0.988–0.995, *p* < 0.001)	0.99 (0.990–0.996, *p* < 0.001)	0.99 (0.990–0.995, *p* < 0.001)	0.99 (0.992–0.996, *p* < 0.001)	0.99 (0.994–0.997, *p* < 0.001)	0.99 (0.995–0.998, *p* < 0.001)
ICC (95% CI, *p* value)	0.99 (0.986–0.994, *p* < 0.001)	0.99 (0.989–0.995, *p* < 0.001)	0.99 (0.988–0.995, *p* < 0.001)	0.99 (0.990–0.995, *p* < 0.001)	0.99 (0.993–0.997, *p* < 0.001)	0.99 (0.993–0.997, *p* < 0.001)
Mean Difference (±SD)	−0.28 ± 1.02	−0.14 ± 0.95	−0.29 ± 0.97	−0.17 ± 0.91	−0.04 ± 0.80	−0.07 ± 0.75
Mean MAE (±SD) (BPM)	0.59 ± 0.88	0.63 ± 0.73	0.67 ± 0.76	0.65 ± 0.66	0.52 ± 0.60	0.51 ± 0.56
Agreement (Bias) (%)	−2.44%	−1.85%	−2.54%	−2.02%	−1.29%	−1.82%
95% LOA	−17.32–12.43%	−17.03–13.33%	−17.92–12.84%	−16.92–12.88%	−12.91–10.34%	−14.61–10.97%
Accuracy (%)	94%	96%	95%	96%	99%	98%
**II. Inter-device Reliability (L vs. R)**						
ICC (95% CI, *p* value)	0.98 (0.982–0.992, *p* < 0.001)		0.99 (0.989–0.995, *p* < 0.001)		0.99 (0.991–0.996, *p* < 0.001)	
Mean Difference (±SD) (BPM)	0.14 ± 1.19		0.12 ± 0.95		−0.03 ± 0.86	
Agreement (Bias) (%)	0.59%		0.51%		−0.53%	
95% LOA (%)	−19.01–20.19%		−12.64–13.67%		−14.24–13.18%	

**Table 3:** Summary of the results of apnea duration regarding accuracy and inter-device reliability on each posture. MAE: Mean Absolute Error, LOA: Limits of Agreement, SD: Standard Deviation, CI: Confidence Interval, L: PneumoWave biosensor on the left side, R: PneumoWave biosensor on the right side.

	45°		90°		180°	
	L	R	L	R	L	R
**I. Accuracy (Reference (10 s) vs. Biosensors)**						
Mean Duration (±SD) (s)	9.01 ± 2.60	9.43 ± 1.76	8.58 ± 2.35	8.70 ± 2.42	9.00 ± 2.35	9.05 ± 2.45
Mean Difference (±SD) (s)	0.99 ± 2.60	0.57 ± 1.76	1.42 ± 2.35	1.30 ± 2.42	1.00 ± 2.35	0.95 ± 2.45
Mean MAE (±SD) (s)	1.63 ± 2.24	1.29 ± 1.32	1.73 ± 2.12	1.68 ± 2.17	1.59 ± 2.00	1.65 ± 2.04
Apnea < 5 s	5%	3%	7%	8%	7%	6%
Apnea ≥ 5 s	95%	97%	93%	92%	93%	94%
**II. Inter-device Reliability (L vs. R)**						
Mean Difference (±SD) (s)	−0.41 ± 2.76		−0.12 ± 2.43		−0.05 ± 2.55	
Agreement (Bias) (s)	−0.41		−0.12		−0.05	
95% LOA (95% CI) (s)	−5.82–4.99		−4.88–4.64		−5.05–4.94	

## Data Availability

Data is available upon request by contacting the corresponding author.

## Supplementary Materials

The following supporting information can be downloaded at: https://www.mdpi.com/article/10.3390/bios16050256/s1, Recruitment Eligibility Criteria, Figure S1. PRISMA flow for summary of the in vivo validation and Figure S2. Bland–Altman plots demonstrating the inter-device reliability between the Left (L) and Right (R) biosensors across the 6–30 BPM range.

## Abbreviations

The following abbreviations are used in this manuscript:

AASM	American Academy of Sleep Medicine
ALS	Advanced Life Support
a.u.	Arbitrary Unit
BPM	Breaths Per Minute
BMI	Body Mass Index
CI	Confidence Interval
COPD	Chronic Obstructive Pulmonary Disease
CSV	Comma-Separated Values
DOC	Direct Observational Count
GDPR	General Data Protection Regulation
g	Acceleration
ICC	Intraclass Correlation Coefficient
L	Left PneumoWave Biosensor
LoA	Limits of Agreement
MAE	Mean Absolute Error
MAVC	Manual Average Visual Count
OSA	Obstructive Sleep Apnea
r	Pearson Correlation Coefficient
R	Right PneumoWave Biosensor
R^2^	Coefficient of Determination
RR	Respiratory Rate
SD	Standard Deviation
UKCA	UK Conformity Assessed
