# Supplementary material for: Assessment of Respiratory Rate and Simulated Apnea Utilizing the PneumoWave Biosensor: In Vitro and In Vivo Validation
Source: Biosensors (Basel). 2026 May 1;16(5):256. doi: 10.3390/bios16050256 (PMC13205080; doi:10.3390/bios16050256)
Supplement: Supplementary file 1 [file biosensors-16-00256-s001.zip › biosensors-4225571-supplementary.pdf]

Assessment of Respiratory Rate and Simulated Apnea Utilizing the PneumoWave Biosensor: In Vitro and In Vivo Validation

Burcu Kolukisa Birgec †, Beyza Toprak † and Alexander Balfour Mullen *

Strathclyde Institute of Pharmacy and Biomedical Sciences, University of Strathclyde, Glasgow G4 0RE, UK; burcu.kolukisa@strath.ac.uk (B.K.B.); beyza.toprak.2022@uni.strath.ac.uk (B.T.)

* Correspondence: a.mullen@strath.ac.uk

† These authors contributed equally to this work.

List of Materials

Recruitment Information 2

Figure S1. PRISMA flow for summary of the in vivo validation 3

Figure S2. Bland-Altman plots demonstrating the inter-device reliability between the Left (L) and Right (R) biosensors across the 6 – 30 BPM range 4

Recruitment Information

Inclusion Criteria

Participants must meet the following criteria:

- Male or female
- Any person aged 18 years or over.
- Able and willing to provide written informed consent in English
- Able to breathe at 6 and 30 BPM for 3 minutes

Exclusion Criteria

Participants will be excluded if they meet any of the following conditions:

- Presence of any significant medical condition that may be aggravated by slow or fast breathing
- Unable to breathe at 6 and 30 BPM for 3 minutes
- Any known chronic disease
- Skin fragility or other dermatological conditions preventing secure device attachment
- Inability to access the right or left chest wall for device placement
- Allergy to medical-grade skin adhesives
- Pregnancy

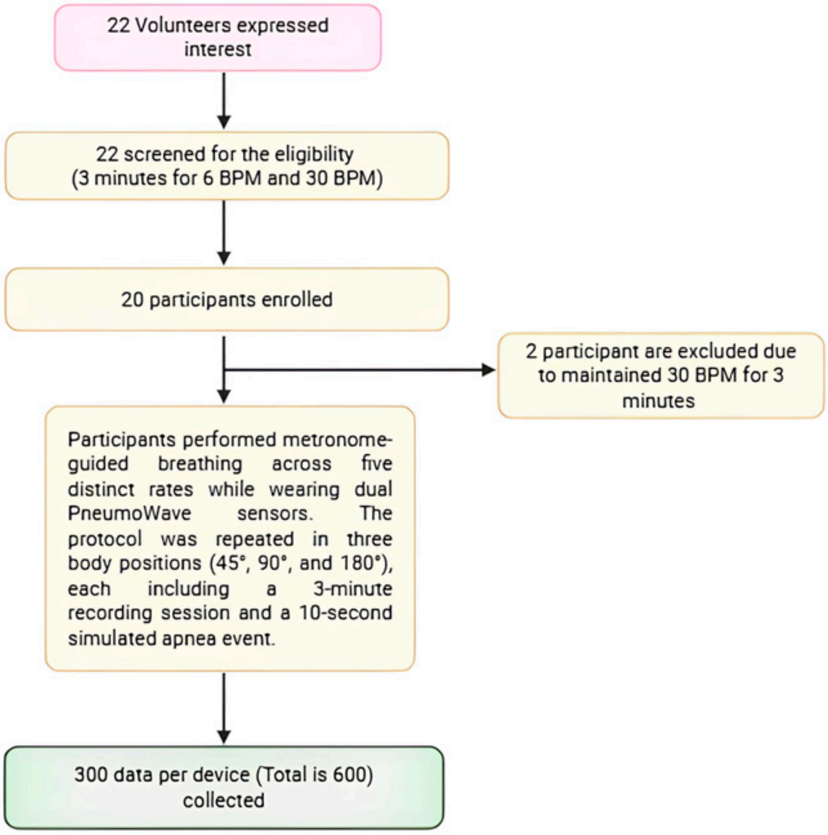

Figure S1. PRISMA flow for summary of the in vivo validation

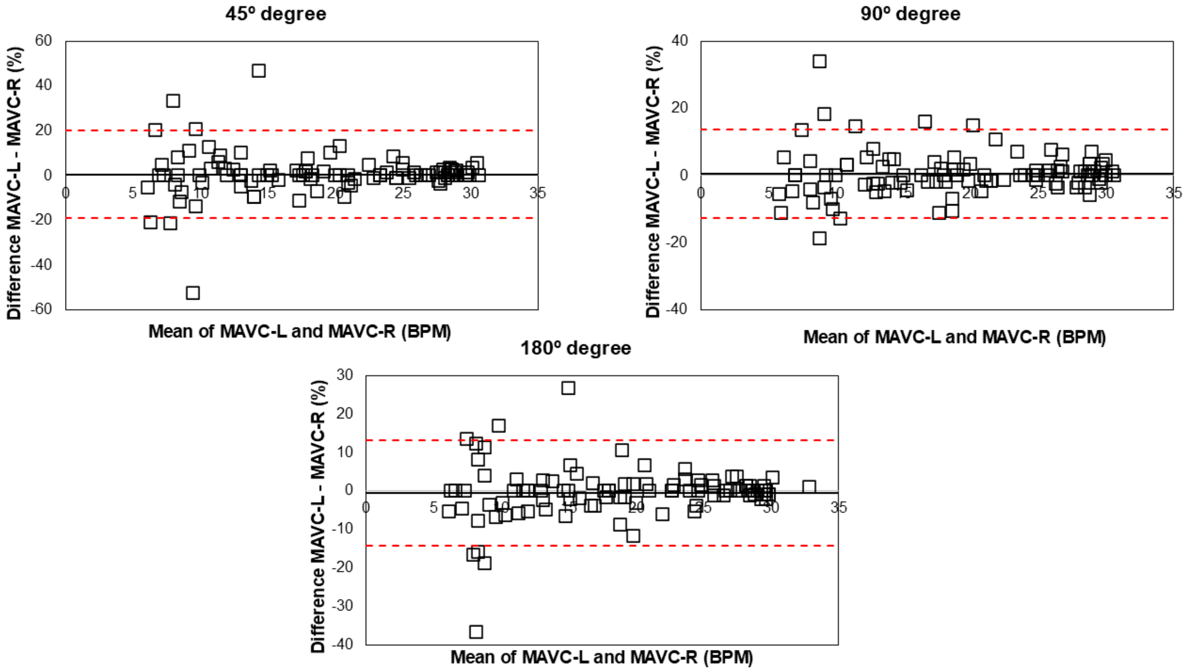

Figure S2. Bland-Altman plots demonstrating the inter-device reliability between the Left (L) and Right (R) biosensors across the 6 – 30 BPM range. Squares represent the percentage difference between the two devices for each measurement. The solid black line indicates the mean bias, while the dashed red lines represent the 95% limits of agreement (± 1.96 SD). MAVC-L: Manual Average Visual Count from PneumoWave biosensor on Left side; MAVC-R: Manual Average Visual Count from PneumoWave biosensor on Right side.
